# Supplementary material for: Management of Adolescents With OUD: A Simulation Case for Subspecialty Trainees in Addiction Medicine and Addiction Psychiatry
Source: MedEdPORTAL. 2021 Apr 20;17:11147. doi: 10.15766/mep_2374-8265.11147 (PMC8056775; doi:10.15766/mep_2374-8265.11147)
Supplement: Supplementary file 1 — OUD Simulation Case.docxDemographic Information Survey.docxConfidence Survey.docxCritical Actions Checklist.docxLearner Packet.docxLearner Satisfaction Survey.docxManagement of Adolescents With OUD.pptStandardized Patient Packet.docxDebriefing Guide.docx [file mep_2374-8265.11147-s001.zip › C. Confidence Survey.docx]

**Appendix C: Confidence Survey: Managing Adolescents with OUD**

After reading the below case, please rate how confident you feel about doing each activity by recording a number between 1 and 10 using the scale provided

1=Cannot do at all 10=Highly certain can do

You are seeing a 16-year-old female (Jessica) in your outpatient office. Jessica has been ordered to treatment by her parole officer. She was arrested for assaulting a police officer while intoxicated and high on heroin. She has been using heroin INH for the past four months.

Establish rapport with this adolescent

1 2 3 4 5 6 7 8 9 10

Explain the limits of confidentiality

1 2 3 4 5 6 7 8 9 10

Identify opioid withdrawal symptoms using the Clinical Opioid Withdrawal Scale (COWS)

1 2 3 4 5 6 7 8 9 10

Rate the severity of opioid withdrawal as mild, moderate, severe

1 2 3 4 5 6 7 8 9 10

Describe at least 3 available treatment options in the state for adolescents with OUD

1 2 3 4 5 6 7 8 9 10

Determine whether it is appropriate to inform the parent/guardian about the substance use behaviors

1 2 3 4 5 6 7 8 9 10
