# Supplementary material for: Management of Adolescents With OUD: A Simulation Case for Subspecialty Trainees in Addiction Medicine and Addiction Psychiatry
Source: MedEdPORTAL. 2021 Apr 20;17:11147. doi: 10.15766/mep_2374-8265.11147 (PMC8056775; doi:10.15766/mep_2374-8265.11147)
Supplement: Supplementary file 1 — OUD Simulation Case.docxDemographic Information Survey.docxConfidence Survey.docxCritical Actions Checklist.docxLearner Packet.docxLearner Satisfaction Survey.docxManagement of Adolescents With OUD.pptStandardized Patient Packet.docxDebriefing Guide.docx [file mep_2374-8265.11147-s001.zip › D. Critical Actions Checklist.docx]

**Appendix D: Critical Actions Checklist: To be completed by facilitator as they observe learner**

**THE ADOLESCENT WITH OPIOID USE DISORDER**

*Did the learners complete the following Critical Actions?*

______________________________________________________________________

- Please make sure this sheet includes the learner ID above
- Please make sure the actor’s ear piece is working prior to starting simulation
- Alert the learner when there are 5 minutes left for the simulation (use microphone)

*Checklist:*

- Developed Rapport
- Defines limits of confidentiality for adolescent
  - Defines the word confidentiality
  - Describes exceptions to confidentiality
  - Describes what would happen if breach of confidentiality is needed
- Notes that patient is in withdrawal
- Uses COWS to assess severity
  - Obtains pulse
  - Observes or asks about chills, sweating
  - Observes or asks about restlessness
  - Checks pupils
  - Asks about bone/joint pain while assessing baseline pain severity
  - Asks about nasal stuffiness, tearing, accounting for potential recent allergies/cold
  - Asks about GI upset within the last ½ hour
  - Asks patient to extend hands/observes and notes tremor
  - Observes for yawning
  - Asks about anxiety and irritability.
  - Checks for piloerection
- Calculates COWS score: Score:_________________________________
- Explains diagnosis OUD
- Assesses motivation to receive ongoing treatment
- Explains medication treatment options to adolescent
  - Symptom Management/ Detox
  - Naltrexone + psychosocial treatment
  - Buprenorphine + psychosocial treatment
  - Methadone
