# Supplementary material for: Management of Adolescents With OUD: A Simulation Case for Subspecialty Trainees in Addiction Medicine and Addiction Psychiatry
Source: MedEdPORTAL. 2021 Apr 20;17:11147. doi: 10.15766/mep_2374-8265.11147 (PMC8056775; doi:10.15766/mep_2374-8265.11147)
Supplement: Supplementary file 1 — OUD Simulation Case.docxDemographic Information Survey.docxConfidence Survey.docxCritical Actions Checklist.docxLearner Packet.docxLearner Satisfaction Survey.docxManagement of Adolescents With OUD.pptStandardized Patient Packet.docxDebriefing Guide.docx [file mep_2374-8265.11147-s001.zip › E. Learner Packet.docx]

Appendix E: Learner Packet

SIM philosophy/ Confidentiality

- The main purpose of this activity is to provide you with an opportunity to practice new skills, gain confidence, and “learn by doing.” The purpose is NOT to test or evaluate you.
- You may feel nervous at times but historically participants really enjoy the Simulation experience.
- Ideally you will identify gaps in your knowledge and formulate questions which can be raised and discussed during the group debrief.
- **Safe space**: “What happens in the Simulation Center stays in the Simulation Center.” It’s best we don’t discuss each other’s performance with colleagues who didn’t attend the Simulation. In the same respect, please refrain from discussing any details of our SPs award winning performances.

Fiction Contract

- We ask you try and suspend disbelief during these exercises. Go ahead, raise the stakes, take risks and really be present in the scene.
- This is your opportunity to step out of your comfort zone and practice new skills in a non-threatening environment

We hope you enjoy your simulation experience! As always, we are here for your education, so please let us know if you have any questions, comments and/or feedback.

Your ID for this Simulation:

- First letter Mother’s maiden name
- First two numbers of your birth month
- First initial middle name

**New patient Intake Form**

Pt name: **Sarah Munson/ William Harris**

Age **17**

Reason for Visit: **Evaluation- requesting treatment**

Insurance: **Medicaid- Active**

Vitals: Pulse: **93** BP: **110/87**  Temp **99.0**

Utox Instant Screen results:

Opiates ______**x**_

Benzos _________

Marijuana ______ **x**

Cocaine _________

Methadone _________

Buprenorphine _________

Breathalyzer: **0.00**

Urine pregnancy test: **Negative**


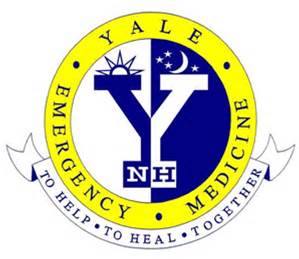


**Scenario Overview**

Sarah Munson/ William Harris is a 17 year old that presents to your outpatient office by referral of the local substance abuse center. The patient is an adolescent and the local center does not treat minors. Luckily there was a cancellation and you were able to schedule the patient that same day. You are aware of the patient’s history which was faxed to you. The patient’s mother is outside your office and the adolescent is waiting to meet with you in private. You introduce yourself and obtain a focused history regarding substance use and perform the relevant focused exam.

**Goals and Objectives**

- The learner will demonstrate how a physician explain confidentiality (as it pertains to substance use care) to a minor with a severe drug use disorder
- The learner will assess the 11 signs/symptoms necessary to complete the Clinical Opioid Withdrawal Scale (COWS)
- The learner will clearly and supportively explain the appropriate treatment options to adolescents with sever Opioid Use Disorder.

Clinical Opiate Withdrawal Scale (COWS)

For each item, write in the number that best describes the patient’s signs or symptom. Rate on just the apparent relationship to opiate withdrawal. For example, if heart rate is increased because the patient was jogging just prior to assessment, the increase pulse rate would not add to the score.

| **Resting Pulse Rate**: (record beats per minute)  *Measured after patient is sitting or lying for one minute*  0 pulse rate 80 or below  1 pulse rate 81-100  2 pulse rate 101-120  4 pulse rate greater than 120 | |  |  |  |  |
| --- | --- | --- | --- | --- | --- |
| **Sweating:** *over past ½ hour not accounted for by room temperature or patient activity.*  0 no report of chills or flushing  1 subjective report of chills or flushing  2 flushed or observable moistness on face  3 beads of sweat on brow or face  4 sweat streaming off face | |  |  |  |  |
| **Restlessness** *Observation during assessmen*t  0 able to sit still  1 reports difficulty sitting still, but is able to do so  3 frequent shifting or extraneous movements of legs/arms  5 Unable to sit still for more than a few seconds | |  |  |  |  |
| **Pupil size**  0 pupils pinned or normal size for room light  1 pupils possibly larger than normal for room light  2 pupils moderately dilated  5 pupils so dilated that only the rim of the iris is visible | |  |  |  |  |
| **Bone or Joint aches** *If patient was having pain previously, only the additional component attributed to opiates withdrawal is scored*  0 not present  1 mild diffuse discomfort  2 patient reports severe diffuse aching of joints/ muscles  4 patient is rubbing joints or muscles and is unable to sit still because of discomfort | |  |  |  |  |
| **Runny nose or tearing** *Not accounted for by cold symptoms or allergies*  0 not present  1 nasal stuffiness or unusually moist eyes  2 nose running or tearing  4 nose constantly running or tears streaming down cheeks | |  |  |  |  |
| **GI Upset**: *over last ½ hour*  0 no GI symptoms  1 stomach cramps  2 nausea or loose stool  3 vomiting or diarrhea  5 Multiple episodes of diarrhea or vomiting |  | |  |  |  |
| **Tremor** *observation of outstretched hands*  0 No tremor  1 tremor can be felt, but not observed  2 slight tremor observable  4 gross tremor or muscle twitching |  | |  |  |  |
| **Yawning** *Observation during assessment*  0 no yawning  1 yawning once or twice during assessment  2 yawning three or more times during assessment  4 yawning several times/minute |  | |  |  |  |
| **Anxiety or Irritability**  0 none  1 patient reports increasing irritability or anxiousness  2 patient obviously irritable anxious  4 patient so irritable or anxious that participation in the assessment is difficult |  | |  |  |  |
| **Gooseflesh skin**  0 skin is smooth  3 piloerrection of skin can be felt or hairs standing up on arms  5 prominent piloerrection |  | |  |  |  |
| **Total scores**  **with observer’s initials** | ­­­­­­­  ­­­­­ | | ­­­­­­­  ­­­­­ | ­­­­­­­  ­­­­­ | ­­­­­­­  ­­­­­ |

**Score:**

**5-12 = mild;**

**13-24 = moderate;**

**25-36 = moderately severe;**

**more than 36 = severe withdrawal**

**Learner Self- Debriefing**

**THE ADOLESCENT WITH OPIOID USE DISORDER**

**Thank you for completing the simulation with the standardized patient. To prepare for the debriefing session with the Faculty Member, please take a few minutes to think about the following questions:**

**1. How did you feel about the case?**

**2. Can you summarize what happened during the case?**

**You will review your thoughts and summaries with the Faculty Member during the Debriefing Session.**

**FOR DEBRIEFING SESSION:**

Learning Goals:

1.

2.

3.
