# Supplementary material for: Management of Adolescents With OUD: A Simulation Case for Subspecialty Trainees in Addiction Medicine and Addiction Psychiatry
Source: MedEdPORTAL. 2021 Apr 20;17:11147. doi: 10.15766/mep_2374-8265.11147 (PMC8056775; doi:10.15766/mep_2374-8265.11147)
Supplement: Supplementary file 1 — OUD Simulation Case.docxDemographic Information Survey.docxConfidence Survey.docxCritical Actions Checklist.docxLearner Packet.docxLearner Satisfaction Survey.docxManagement of Adolescents With OUD.pptStandardized Patient Packet.docxDebriefing Guide.docx [file mep_2374-8265.11147-s001.zip › F. Learner Satisfaction Survey.docx]

ID:_________________

**Appendix F: Learner Post Training Satisfaction Survey**

1. Did you find the exercise effective? Yes No
2. Would you recommend the training to others? Yes No
3. Did you feel comfortable during the training? Yes No
