# Supplementary material for: Management of Adolescents With OUD: A Simulation Case for Subspecialty Trainees in Addiction Medicine and Addiction Psychiatry
Source: MedEdPORTAL. 2021 Apr 20;17:11147. doi: 10.15766/mep_2374-8265.11147 (PMC8056775; doi:10.15766/mep_2374-8265.11147)
Supplement: Supplementary file 1 — OUD Simulation Case.docxDemographic Information Survey.docxConfidence Survey.docxCritical Actions Checklist.docxLearner Packet.docxLearner Satisfaction Survey.docxManagement of Adolescents With OUD.pptStandardized Patient Packet.docxDebriefing Guide.docx [file mep_2374-8265.11147-s001.zip › H. Standardized Patient Packet.docx]

| **Appendix H: Standardized Patient Packet**  **Simulation Case Title: Management of Adolescents with OUD** | |
| --- | --- |
| **Author** | Deepa Camenga, MD, MHS |
| **Date** | August 10, 2016 |
| **Audience** | Addiction Psychiatry and Medicine Fellows or Trainers of Residents of Students |
| **Brief Narrative Description of the Case** | William Harris (or Sarah Munson) is a 17 yo male who presents with his mother to your office/ED as he has just told his mom that he is using heroin (last use about 12 hours ago and pt is in withdrawal). He has a 9 month h/o inhaled heroin use in total, and has used IV heroin several times recently (currently ~3-5 bags/day). He has not told his mother about his IVDU, but mom is aware of inhaled heroin use.  The learner should demonstrate proficiency in describing the limits of confidentiality (for substance use care) to adolescent, demonstrate proficiency in using the COWS/Clinical Opioid Withdrawal and be able to describe at least 3 treatment options to an adolescent with OUD |
| **Learning Objectives**  **Critical Actions** | **Goals and Objectives:**  By the end of this activity, learners will be able to:  1. Demonstrate understanding of local confidentiality laws (as it pertains to substance use care) by explaining the concepts to a minor with a severe opioid use disorder  2. Assess for the presence of opioid withdrawal by using the Clinical Opiate Withdrawal Scale (COWS)  3. Explain age-appropriate treatment options (including behavioral support without medications, buprenorphine, and naltrexone) to adolescents with severe opioid use disorder using patient-centered communication strategies   1. Define limits of confidentiality (meaning, exceptions, breach) 2. Perform critical parts of history necessary to diagnose opioid withdrawal 3. Assess for pt. for feelings of chills/flushing 4. Assess whether patient is experiencing Nausea/vomiting/diarrhea 5. Assess whether patient is experiencing feelings of anxiety/irritability 6. Assess whether the patient is experiencing pain 7. Assess resting pulse rate after patient is sitting or lying 8. Assess pupil size 9. Assess for tremor by asking patient to show outstretched hands 10. Assess arms for piloerection 11. Assess patient for runny nose/tearing 12. Identify restlessness in patient 13. Observe patient for yawning 14. Demonstrate ability to identify that patient is in mild withdrawal 15. Explain treatment options to the adolescent (symptom management/detox, psychosocial treatment, medications of OUD) |
| **Learner Preparation** | One-week prior, learners view a 10-minute PowerPoint video addressing learning objectives. The day of the exercise, trainees received the learner packet which included the simulation-based learning philosophy, case description, learning objectives, patient vital signs, urine toxicology results, Clinical Opiate Withdrawal Scale (COWS) scale, and the self-debrief. |

| **Overall Setting and Appearance** | The scene takes place in an outpatient evaluation room. There is an evaluation table for the patient and a chair for the trainee. The actor resembles the age of an adolescent and appears in mild withdrawal. |  |
| --- | --- | --- |
| **Patient Information** | **Name:** William Harris or Sarah Munson  **Gender:** natal male or female, identify as cis-gender  **Patient Demeanor**: You are a quiet teenager but answer questions honestly and openly. You are interested in getting treatment and if the provider seems supportive and trustworthy, can open up and be thankful for the care you receive.  However, if the medical provider “talks down to you” or offends you, though, you tend ot resist talking more and resort to saying more vague statements like “I don’t know” or “Kind of” or “Sure, whatever”. | **Age:** 17  **Weight:** N/A  **Medications:** None  **Allergies:** None |
| **Props** | The standardized patient should check their cell phone frequently throughout the visit. If they have a bag, they should often look through it looking for their phone or other items | |
| **Key points** | You are a 17-year-old male that presents to the Addiction Doctor by referral of the local drug treatment center, as you are a minor and they do not treat minors. In order to move the case along, you should ask the following questions to the doctor to help them get through the case:   1. Early on you will be concerned about privacy. You will ask “Are you going to tell my mom about the IV, she doesn’t know I use IV heroin, I only told her I snort it” 2. After the doctor gets the history they need to assess you for signs of withdrawal, you can prompt them to do this by stating “I need to smoke a cigarette and walk around- when is this going to end.” 3. After they have done an exam, you should state “So what treatment do you guys offer here” and “what kind of treatment do you think I should have”   If the doctor asks to speak with your mom, you state   1. “she texted me and said she had to drop my grandma off at dialysis, she’ll be back if you need me to text her”   If the doctor is not finishing up the case well, you can state   1. I need to go, my boyfriend/girlfriend is waiting for me. | |
| **SUMMARY OF YOUR DISCUSSION WITH THE DRUG TREATMENT CENTER COUNSELOR:**  **Backstory (for actors’ eyes only)** | | |
| **HPI/ROS:**  **Chief Complaint**  **Past Medical history, Current Medications or Drug Allergies**  **Past Psychiatric history**  **Substance Use History**  **Overdose history**  **Use of illicit methadone/buprenorphine**  **Developmental history**  **Educational History**  **Home Environment**  **Romantic relationships**  **Strengths**  **Future Orientation** | **Counselor**: How can we help you today?  *Chief complaint: “****I am sick of being sick every day****”*  **William**: “I am sick of being sick every day so I finally broke down and called my mom. I started using Percocet about 1 year and a half ago. My friend and I got them from his older brother, I then started sniffing heroin a few months ago (9 months ago) and for the past few months started to do IV with my girlfriend. I do up to 3-5 bags a day. One of my friends got some bad stuff so I tried to stop but couldn’t because I got dope sick. I finally ran out of money to buy and broke down and told my mom this morning. I am sick of waking up every day trying to figure out where to get drugs. I’m tired of it. I talked to my friend who is in treatment and she suggested I told my mom. My mom cried when I told her but she googled something and made me talk to someone in my town. They sent me here.”  **Counselor**: Do you have any medical conditions? Are you on any medications or have any allergies?  *PMH/Meds/Allergies- none*  **William** “No, but…  *Psych history*: “I was in the hospital at the end of middle school. I said I was going to hang myself and was hospitalized for a few days when I was in 8^th^ grade. Then my parents put me in a rehab soon after that (at Rushford, for daily marijuana use and depression) for 2 weeks and I went back to start high school. I saw a therapist a few times in 9^th^ grade” but then we lost our insurance for a while and I stopped going. I also was diagnosed with ADHD age 8, treated by my pediatrician with Ritalin then Concerta until age 15.  **Counselor**: I am now going to ask about other drugs, alcohol and tobacco use...  **William**:   - CIGARETTES: I smoke 5 cigarettes per day and started at age 13. I tried e-cigarettes a couple of times - ALCOHOL: I drink 2-4 beers about two weekends a month. I drink to get drunk and have never passed out. I started to try it at age 13 but didn’t get drunk until I was about 14 - MARIJUANA: I smoke marijuana about 3-5 times per week and first smoked around age 13   **Counselor**: Have you ever had an overdose? Or tried methadone or suboxone before?  **William**:   - I have never overdosed on heroin. - I have never tried methadone, but don’t hear good things about it. I tried suboxone strips on the street a few weeks ago, but they are hard to find.   *Developmental history* (per mother): “William was a full-term baby and I had a natural birth (vaginal delivery). He was healthy when he was born, but I smoked cigarettes in first and second trimester and started again pretty soon after the birth. He walked and talked at the normal time and he had some trouble reading in elementary school.”  **Counselor**: How did you do in school? How about now?  *Educational history*:  **William**: “I repeated kindergarten because I had some trouble with reading because of the ADHD. I started high school but didn’t get along with some of the kids and was suspended a lot- so transferred to a technical school in 10^th^ grade. I stopped going a few months ago but am trying to figure out how to get a GED.”  **Counselor**: What are things like at home?  **William**: “I don’t know where my dad is and my mom and dad broke up when I was 2. I don’t really remember him. I used to live with my grandma in elementary school, until I was about 12 years old, because my mom was struggling with money and lived in another state for a while. She came back when I was 12 after she married my stepfather. I live with my mom and my stepfather now. My mom works in a salon and my dad works at a golf course cutting the lawn. My grandma’s on dialysis now and mom spends a lot of time taking care of her and driving her to dialysis so can’t work too much.”  **Counselor**: Do you have any siblings?  **William** “My brother Tom moved out when he was 17. I think he lives in North Carolina. I don’t really talk to him except for Facebook sometimes.”  **Counselor**: Are you in a relationship?  **William**: “I have been with my girlfriend, Monica, for a while. She helped me figure out how to talk to my mom because she has been seeing a psychiatrist for depression and stuff. Monica uses but is trying to cut down and stop”.  **Counselor**: What are some qualities about yourself that you think will help you succeed in treatment?  **William** “I don’t know. I can make friends easily and my friends tell me I am funny and nice. My girlfriend says that I treat her well too and we are trying to get clean together.”  **Counselo**r: What do you see yourself doing in a year?  **William**: I hope to get clean. Then I want to find a job so I can buy a car and eventually move out of my mom’s house. | |
|  |  | |
| **Abnormal Physical Exam Findings** | You feel slightly sick to your stomach and nervous/fidgety  You yawn several times during the interview  You rub your nose/sniffle several times during the interview  Your back hurts so you rub it a bit and fidget when you are sitting  If you doctor asks to see your arms, you point out that you have needle marks (track marks) on your right arm only. You also have a very mild tremor if the doctor asks you to stretch your hands out. | |

Set up: Need exam table (patient will sit on edge of it) and chair next to the exam table.

| **Duration or Event Based** | **Monitor Settings** | **Patient Auscultation sounds** | **Patient Parameters** | **Trigger/Cue to next state** |
| --- | --- | --- | --- | --- |
| **Initial State** | **HR:** 90-100  **ECG Rhythm:**  **RR: 20**  **BP: 110/68**  **Sat: 99**  **ETCO2:**  **Temp: 98.8** | **Heart; Tachy**  **Lung: Clear**  **Bowel: Nl** | **Eyes: pupils appropriate for room lighting**  **Convulsions: Mild hand tremor** |  |

| **Laboratory Results** | **Na:**  **K:**  **CL:**  **HCO3:**  **BUN:**  **Cr:**  **Glucose:**  **Lipase:**  **AST:**  **ALT:**  **T.Bili:**  **D.Bili:** | **Utox: + opiates, marijuana neg benzos,cocaine etc. breathalyzer 0**  **UPT: negative** | **pH:**  **paCO2:**  **paO2:**  **FIO2:**  **EtOH:** |  |
| --- | --- | --- | --- | --- |
| **Radiology/Media Results NONE** | | | | |
| **Medication to be available: Naloxone** | | | | |

**CHEAT SHEET FOR SP**

Pt name: **Sarah Munson or William Harris**

Age **17**

Reason for Visit: **Evaluation- requesting treatment**

Your symptoms include:

Mild feelings of chills/flushing (you feel this but don’t exhibit any physical evidence of this)

Mild stomach pain (3/10 on pain scale)

Mild feelings of anxiousness

Leg pain (aching in my leg bones 4/10 on pain scale- no physical evidence of this on exam)

Mild difficulty sitting still—look in your bag or at your phone a lot
