# Supplementary material for: Management of Adolescents With OUD: A Simulation Case for Subspecialty Trainees in Addiction Medicine and Addiction Psychiatry
Source: MedEdPORTAL. 2021 Apr 20;17:11147. doi: 10.15766/mep_2374-8265.11147 (PMC8056775; doi:10.15766/mep_2374-8265.11147)
Supplement: Supplementary file 1 — OUD Simulation Case.docxDemographic Information Survey.docxConfidence Survey.docxCritical Actions Checklist.docxLearner Packet.docxLearner Satisfaction Survey.docxManagement of Adolescents With OUD.pptStandardized Patient Packet.docxDebriefing Guide.docx [file mep_2374-8265.11147-s001.zip › I. Debriefing Guide.docx]

**Appendix I: Debriefing Guideline**

**THE ADOLESCENT WITH OPIOID USE DISORDER**

______________________________________________________________________

**Part 1: Self-Debrief (1-2 minutes):** *(Send Learner immediately following scenario to the debriefing room. Tell the Learner to initiate the Self-Debriefing for Case.)*

**Part 2: Begin Self-Debrief: 1-2 minutes)**

- How did you feel about the case?
- Can you summarize what happened?
- **Part 2a: Learner Reviews Self-Debrief with the Faculty Member**

**Part 3: Trainer Reviews Medical/Clinical Knowledge with Learner (15 minutes).**

*This case was designed to review three major learning objectives: 1) demonstrate understanding of confidentiality laws by explaining the concepts to a minor with OUD 2) assess for the presence of opioid withdrawal by using the COWS scale 3) explain age appropriate medication treatment options to adolescents with OUD*

1. **Trainer Reviews Adolescent Confidentiality: CT Specific Laws**

**Teaching Points:**

- Parental consent is necessary for medical or surgical treatment of minors, except in:
  - Reproductive healthcare, treatment of drug or alcohol abuse, certain mental healthcare needs
  - If the minor is emancipated.
    - Definition: 16 or 17 year old’s who have obtained a court order because the minor has been married, actively serves in the U.S. Armed Forces, willingly lives away from home with or without parental consent and manages his or her own finances, the court determines “for good cause” that emancipation is in the “best interest” of the minor.

Exceptions include:

- - During an emergency when it is either impractical to obtain parental consent or any delay would unduly endanger the patient’s life.
  - When there is a serious threat to the **incompetent** minor’s life or physical well-being and it is determined that this threat can be diminished by disclosure to the parents.

Questions that inform decision-around confidentiality

- - Does this teen have a right to confidentiality for their specific healthcare complaint?
  - Are they presenting with an emergency where it is impractical to obtain consent/it would delay life-saving treatment?
  - Is there a serious threat to incompetent minors’ life or physical well-being?

1. **Trainer review COWS assessment, use critical actions checklist for the learner’s management**

**Teaching Points:**

1. To complete the COWS, the physician must perform critical parts of history necessary to diagnose opioid withdrawal
2. Assess for pt. for feelings of chills/flushing (SCORE =1: subjective reports of chills)
3. Assess whether patient is experiencing Nausea/vomiting/diarrhea (SCORE=1 for subjective report of stomach pain)
4. Assess whether patient is experiencing feelings of anxiety/irritability (pt. will state I need to smoke a cigarette and also respond yes: SCORE=1)
5. Assess whether the patient is experiencing pain: (pt. will state my legs hurt 4/10 on pain scale, “its aching in my leg bones”.
6. To complete the COWS, the physician must perform critical parts of physical exam necessary to diagnose opioid withdrawal
7. Assess resting pulse rate after patient is sitting or lying for a t least 1 minute (pulse=92 SCORE=2)
8. Assess pupil size (normal for room lighting; SCORE=0)
9. Assess for tremor by asking patient to show outstretched hands (mild tremor can be felt, (SCORE=1)
10. Assess arms for piloerection (no piloerection SCORE=0)
11. Assess patient for runny nose/tearing (none SCORE=0)
12. Identify restlessness in patient (pt. will exhibit mild difficulty sitting still; SCORE=1)
13. Observe patient for yawning- (no yawning SCORE=0)
14. To complete the COWS, the physician must demonstrate ability to identify that patient is in mild withdrawal (TOTAL SCORE =7)

**3. Trainer reviews treatment options for the adolescent**

**Teaching Points:**

- Language: It’s helpful to use language which the adolescent understands instead of medical jargon
- Encourage patients to tell parents about their use and treatment in order to increase support network which might be helpful with treatment adherence, transportation to care and coordination of treatment with other activities such as school or work.
- Discuss treatment in terms of risks and benefits
- Some families will not be open to agonist/partial agonist treatment
- Naltrexone: FDA label 18 although some observational studies indicate younger people can benefit from long acting preparation. There are no large-scale clinical trials testing the efficacy of naltrexone in adolescents.
- Buprenorphine: FDA labeled 16 and above. Three RCTs exist for adolescents. Trials demonstrate improvement in opioid use and improved treatment retention among youth randomized to the longer course of buprenorphine.
- Methadone: For patients younger than 18 admission criteria to OTP are different (opioid addicted and 1 year hx of addiction). Patients younger than 18 also require:
  - 2 documented, unsuccessful, medically supervised withdrawals or treatment without MAT in a 12 month period.
  - Written parental/guardian consent
  - Not all states allow methadone be dispensed to minors
