# Supplementary material for: Management of Adolescents With OUD: A Simulation Case for Subspecialty Trainees in Addiction Medicine and Addiction Psychiatry
Source: MedEdPORTAL. 2021 Apr 20;17:11147. doi: 10.15766/mep_2374-8265.11147 (PMC8056775; doi:10.15766/mep_2374-8265.11147)
Supplement: Supplementary file 1 — OUD Simulation Case.docxDemographic Information Survey.docxConfidence Survey.docxCritical Actions Checklist.docxLearner Packet.docxLearner Satisfaction Survey.docxManagement of Adolescents With OUD.pptStandardized Patient Packet.docxDebriefing Guide.docx [file mep_2374-8265.11147-s001.zip › A. OUD Simulation Case.docx]

| **Appendix A: Simulation Case**  SIMULATION CASE TITLE: Diagnosing and Treating Adolescents with OUD  **AUTHORS:** Deepa Camenga, MD, MHS  **LEARNER AUDIENCE:** Addiction Psychiatry and Medicine Fellows or Trainers of Residents of Students | |
| --- | --- |
| **PATIENT NAME:** William Harris (or Sarah Munson)  **PATIENT AGE:** 17  **CHIEF COMPLAINT:** "I am sick of being sick every day."  **PHYSICAL SETTING:** Outpatient healthcare office | |
|  | |
| **Brief narrative description of case** | William Harris (or Sarah Munson) is a 17 y/o male who presents with his mother to your office as he has just told his mom that he is using heroin (last use about 12 hours ago and patient is in withdrawal). He has a 9 month history inhaled heroin use in total, and has used IV heroin several times recently (currently ~3-5 bags/day). He has not told his mother about his IVDU, but mom is aware of inhaled heroin use.  The learner should demonstrate proficiency in describing the limits of confidentiality (for substance use care) to adolescent, demonstrate proficiency in using the COWS/Clinical Opioid Withdrawal and be able to describe at least 3 treatment options to an adolescent with OUD |
| **Primary Learning Objectives** | By the end of this activity, learners will be able to:  1. Demonstrate understanding of local confidentiality laws (as it pertains to substance use care) by explaining the concepts to a minor with a severe opioid use disorder  2. Assess for the presence of opioid withdrawal by using the Clinical Opiate Withdrawal Scale (COWS)  3. Explain age-appropriate treatment options (including behavioral support without medications, buprenorphine, and naltrexone) to adolescents with severe opioid use disorder using patient-centered communication strategies |
| **Critical Actions** | 1. Define limits of confidentiality (meaning, exceptions, breach) 2. Perform critical parts of history necessary to diagnose opioid withdrawal 3. Assess for pt. for feelings of chills/flushing 4. Assess whether patient is experiencing Nausea/vomiting/diarrhea 5. Assess whether patient is experiencing feelings of anxiety/irritability 6. Assess whether the patient is experiencing pain 7. Assess resting pulse rate after patient is sitting or lying 8. Assess pupil size 9. Assess for tremor by asking patient to show outstretched hands 10. Assess arms for piloerection 11. Assess patient for runny nose/tearing 12. Identify restlessness in patient 13. Observe patient for yawning 14. Demonstrate ability to identify that patient is in mild withdrawal   4. Explain treatment options to the adolescent symptom management/detox, psychosocial treatment, medications of OUD) |
| **Learner Preparation or Prework** | One-week prior, learners view a 10-minute PowerPoint video addressing learning objectives. The day of the exercise, trainees received the learner packet which included the simulation-based learning philosophy, case description, learning objectives, patient vital signs, urine toxicology results, Clinical Opiate Withdrawal Scale (COWS) scale, and the self-debrief. |

| Initial Presentation | | | |
| --- | --- | --- | --- |
| **Initial vital signs** | HR: 90-100, RR: 20, BP: 110/68, Temp: 98.8, Sat: 99 | | |
| **Overall Setting and Appearance** | The scene takes place in an outpatient evaluation room. There is an evaluation table for the patient and a chair for the trainee. The actor resembles the age of an adolescent and appears in mild withdrawal | | |
| **Confederates (e.g., standardized participants) and their roles in the room at case start** | There are no confederates in this case. | | |
| **HPI** | *Chief complaint: “I am sick of being sick every day”*  HPI: “I am sick of being sick every day so I finally broke down and called my mom. I started using Percocet about 1 year and a half ago. My friend and I got them from his older brother, I then started sniffing heroin a few months ago (9 months ago) and for the past few months started to do IV with my girlfriend. I do up to 3-5 bags a day. One of my friends got some bad stuff so I tried to stop but couldn’t because I got dope sick. I finally ran out of money to buy and broke down and told my mom this morning. I am sick of waking up every day trying to figure out where to get drugs. I’m tired of it. I talked to my friend who is in treatment and she suggested I told my mom. My mom cried when I told her but she googled something and made me talk to someone in my town. They sent me here.” | | |
| **Past Medical/Surgical History** | **Substance Use History** | **Allergies/Medications** | **Family History** |
| “I was in the hospital at the end of middle school. I said I was going to hang myself and was hospitalized for a few days when I was in 8^th^ grade. Then my parents put me in a rehab soon after that for 2 weeks and I went back to start high school. I saw a therapist a few times in 9^th^ grade but then we lost our insurance for a while and I stopped going. I also was diagnosed with ADHD at age 8, treated by my pediatrician with Ritalin then Concerta until age 15." | CIGARETTES: "I smoke 5 cigarettes per day and started at age 13. I tried e-cigarettes a couple of times."  ALCOHOL: "I drink 2-4 beers about two weekends a month. I drink to get drunk and have never passed out. I started to try it at age 13 but didn’t get drunk until I was about 14."  MARIJUANA: "I smoke marijuana about 3-5 times per week and first smoked around age 13."  "I have never overdosed on heroin."  "I have never tried methadone, but don’t hear good things about it. I tried suboxone strips on the street a few weeks ago, but they are hard to find." | NKDA  No current medications | "My father left when I was two and I don’t know much about him. My grandmother is on dialysis and my mom has to take care of her. I have a brother in North Carolina but we don’t keep in touch." |
| **Physical Examination** | | | |
| **General** | Yawns several times during the interview | | |
| **HEENT** | Dilated pupils, runny nose, tearing | | |
| **Neck** | Complains of neck and muscle pain | | |
| **Lungs** | Clear | | |
| **Cardiovascular** | WNL | | |
| **Abdomen** | Complains of stomach cramps | | |
| **Neurological** | Mild tremor | | |
| **Skin** | Gooseflesh skin (piloerection), needle marks on right antecubital fossa, no signs of infection | | |
| **GU** | WNL | | |
| **Psychiatric** | Nervous, fidgety, irritable at times | | |

| Instructor Notes - Changes and CASE Branch Points | | |
| --- | --- | --- |
| **Intervention / Time point** | **Change in Case** | **Additional Information** |
| *Few minutes into the case* | Early on you will be concerned about privacy. You will ask “Are you going to tell my mom about the IV, she doesn’t know I use IV heroin, I only told her I snort it” | *None* |
| *After the learner completes taking the history* | Lerner needs to assess you for signs of withdrawal, you can prompt them to do this by stating “I need to smoke a cigarette and walk around- when is this going to end.” | Patient will pretend to have runny nose (wipe his nose), chills (shiver, put on sweatshirt), muscle and bone pain (complain of aches and pains, rub back), hold their stomach and complain of nausea, pretend to have mild tremor in hands, yawn, become irritable at times, fidget |
| *After the learner completes assessment of withdrawal* | After they have done an exam, you should state “So what treatment do you guys offer here” and “what kind of treatment do you think I should have” |  |
| *If the learner asks to speak with patient's mom* | You state, “She texted me and said she had to drop my grandma off at dialysis, she’ll be back if you need me to text her” |  |
| If the learner is taking too long | You state, "I need to go, my girlfriend is waiting for me." |  |

**Ideal Scenario Flow**

*The provider enters the room to find an adolescent patient sitting on the examination table. The adolescent is fidgety and anxious but cooperative. The provider explores the adolescent's chief complaint as they build rapport and explain local confidentiality laws. The provider recognizes the patient is in opioid withdrawal and utilizes the COWS scale to assess severity, noting active mild withdrawal. After completing the physical examination and obtaining an appropriate history, the provider explains Opioid Use Disorder and assesses patient's motivation to receive ongoing treatment. The provider explains treatment options to the adolescent using language the patient can understand.*

**Anticipated Management Mistakes**

1. *Difficulty with time management: We found some learners ran out of time and were unable to complete the assessment. We included a large clock in the room which counted down the time. Once the clock was incorporated learners were able to complete the assessment in 20 minutes or less.*
2. *Actor and learners were unclear when to end the scene: Some of our learners and actors did not immediately recognize when the learning objectives were met and were unclear when to end the scene. We found that knocking on the door and stating "your next patient is here" was a helpful prompt and allowed for scene closure.*
3. *Uncertainty about case details: Some actors forgot minor case details during the scene. We incorporated the use of a handheld transceiver to the SP's earpiece in order to provide synchronous case details as the interview unfolded.*
