# Supplementary material for: Management of Adolescents With OUD: A Simulation Case for Subspecialty Trainees in Addiction Medicine and Addiction Psychiatry
Source: MedEdPORTAL. 2021 Apr 20;17:11147. doi: 10.15766/mep_2374-8265.11147 (PMC8056775; doi:10.15766/mep_2374-8265.11147)
Supplement: Supplementary file 1 — OUD Simulation Case.docxDemographic Information Survey.docxConfidence Survey.docxCritical Actions Checklist.docxLearner Packet.docxLearner Satisfaction Survey.docxManagement of Adolescents With OUD.pptStandardized Patient Packet.docxDebriefing Guide.docx [file mep_2374-8265.11147-s001.zip › B. Demographic Information Survey.docx]

**Appendix B: Demographic Information and Previous Training Experience Survey**

What is your Sex?

- Female
- Male

How do you describe your race? (Select all that apply)

- Asian/Pacific Islander
- Black
- White
- American Indian
- Other
- Prefer not to answer

Do you describe yourself as Hispanic or Latino/Latina?

- Yes
- No

Have you had training and/or experience in the diagnosis and treatment of adolescent substance use above and beyond required rotations during residency and fellowship?

- Yes
- No

If you answered yes, which type of training/experiences in the diagnosis and treatment of adolescent substance use have you had? (Select all that apply)

- I completed elective rotations during residency
- I completed elective rotations during fellowship
- I have worked with adolescents with substance use as an attending physician
- I have completed online training about adolescent substance use that were not required by residency/fellowship
- I have attended conferences that were not required by residency/fellowship where I learned about adolescent substance use
- Other:
