# Supplementary material for: Management of Adolescents With OUD: A Simulation Case for Subspecialty Trainees in Addiction Medicine and Addiction Psychiatry
Source: MedEdPORTAL. 2021 Apr 20;17:11147. doi: 10.15766/mep_2374-8265.11147 (PMC8056775; doi:10.15766/mep_2374-8265.11147)
Supplement: Supplementary file 1 — OUD Simulation Case.docxDemographic Information Survey.docxConfidence Survey.docxCritical Actions Checklist.docxLearner Packet.docxLearner Satisfaction Survey.docxManagement of Adolescents With OUD.pptStandardized Patient Packet.docxDebriefing Guide.docx [file mep_2374-8265.11147-s001.zip › Educational_Summary_Report_11147.pdf]

MedEdPORTAL® | The AAMC Journal of Teaching and Learning Resources

Original Publication Open Access

Management of Adolescents With OUD: A Simulation Case for Subspecialty Trainees in Addiction Medicine and Addiction Psychiatry

Gabriela Garcia-Vassallo, MD*, Ellen Lockard Edens, MD, MPE, Brady Heward, MD, Marc A. Auerbach, MD, MSci, Ambrose H. Wong, MD, MSEd, Deepa Camenga, MD, MHS

*Corresponding author: maria.garcia@yale.edu

Abstract

Introduction: The opioid epidemic impacts all ages, yet few published medical education curricula exist to train physicians on how to care for opioid use disorder (OUD) in adolescents, a developmental stage where confidentiality protection is appropriate and contributes to quality health care. We developed a simulation-based educational intervention to increase addiction medicine and addiction psychiatry trainees' confidence in managing adolescents with OUD. **Methods:** Trainees completed a confidence survey and viewed an educational video covering state-specific confidentiality laws pertinent to treating adolescents with OUD. One week later, trainees participated in a simulated encounter where they described the scope of confidentiality to a trained actor, used the Clinical Opiate Withdrawal Scale to assess symptoms of opioid withdrawal, and explained adolescent-specific OUD medication treatment options. Immediately afterward, trainees completed a self-reflection and satisfaction survey and participated in a debriefing session with a faculty member where they identified learning goals. One month later, they completed the confidence survey to quantify changes in confidence. **Results:** Thirty-five fellows (21 male, 14 female) completed the simulation-based educational intervention between 2016 and 2019. When asked to answer yes or no, 96% of participants described the exercise as effective and 100% ($n = 26$) would recommend it to peers. In addition, learners identified future learning goals, including researching specific topics and seeking out additional opportunities to evaluate adolescents with OUD. **Discussion:** Based on our participants' report, this simulation-based educational intervention is an effective teaching method for increasing trainee confidence in managing adolescents with OUD.

Keywords
Adolescent Medicine, Opioid Use Disorder, Simulation, Standardized Patient, Substance Abuse/Addiction, Ethics/Bioethics, Confidentiality, Opioids, Addiction

Educational Objectives

By the end of this activity, learners will be able to:

1. Demonstrate understanding of confidentiality laws (as they pertain to substance use care) by explaining the concepts to a minor with a severe opioid use disorder.
2. Assess for the presence of opioid withdrawal by using the Clinical Opiate Withdrawal Scale.
3. Explain age-appropriate medication treatment options (including buprenorphine, naltrexone, and methadone) to adolescents with severe opioid use disorder using patient-centered communication strategies.

Citation:
Garcia-Vassallo G, Edens EL, Heward B, Auerbach MA, Wong AH, Camenga D. Management of adolescents with OUD: a simulation case for subspecialty trainees in addiction medicine and addiction psychiatry. *MedEdPORTAL.* 2021;17:11147.
https://doi.org/10.15766/mep_2374-8265.11147

Introduction

National rates of hospitalizations and deaths from opioid overdoses in adolescents have risen over the past decade.[1,2] Unfortunately, only a small percentage of adolescents diagnosed with opioid use disorder (OUD) receive evidence-based treatments for their medical condition.[3] A recent multistate cohort study of 4,837 youths with OUD determined one of 21 adolescents younger than 18 years and one of four young adults ages 18-22 years received medication for OUD within 3 months of diagnosis.[4] Americans of all ages encounter a lack of available resources. The 2017 National Survey on Drug Use and Health estimated that over 70% of people who needed treatment for OUD did not receive it.[5] This treatment gap may be partly due to a lack of skills and medical knowledge among treating

Copyright © 2021 Garcia-Vassallo et al. This is an open-access publication distributed under the terms of the Creative Commons Attribution license.

physicians, particularly in the management of adolescents with OUD.[6] Many factors, such as availability of skilled providers, accessibility of clinical sites, and source of payment, determine whether adolescents receive necessary, high-quality health care. Confidentiality is another key factor for this age group because concerns about privacy influence whether many minors will seek health care and openly communicate with health care professionals.[7] A lack of knowledge of minor consent laws amongst medical trainees poses a barrier to offering high-quality health care for this population[8] and speaks to the need to develop more training in this area. Medical training has traditionally lacked a rigorous approach to education about the prevention, identification, and treatment of OUD.[9] Although curricula in peer-reviewed journals such as *MedEdPORTAL* cover medical education topics like opioid risk mitigation strategies,[10] urine drug-screen interpretations,[11] and overdose resuscitation,[12] there are few published curricula targeting adolescent OUD. A recent assessment of progress and needs in addiction medicine training in North America noted few programs focusing on adolescent populations.[13] The widespread implementation of trainee education on adolescent addiction treatment is further inhibited by limited access to faculty who are adolescent addiction specialists, a generally low volume of adolescent patients, and the low availability of adolescent treatment training sites in many communities. To overcome these limitations, it is necessary to create education modules on this topic that can be easily disseminated to trainees across different medical specialties.

The implementation of simulation-based scenarios could address this education gap in adolescent addiction training and prove a useful tool for disseminating information to physicians in training. When used in medical education, simulation improves medical knowledge and confidence in patient-centered conversations that involve complex interpersonal skills, including error disclosure and breaking bad news.[14,15] When simulation training is conducted under the right conditions and outcomes are measured by valid and reliable instruments, research has shown not only that graduate trainees can obtain desired skills in a controlled, simulated environment[16] but also that these skills can transfer to the clinical setting.[17]

We developed a simulation-based educational intervention aimed at increasing addiction medicine and addiction psychiatry trainees' confidence in the management of adolescents with OUD. The simulation focuses on three topics specific to adolescent care, including the understanding of unique aspects of confidentiality, the need to distinguish between mild and more severe forms of OUD, and the ability to communicate age-appropriate medication treatment options to a young patient. We chose to use simulation as our training modality for several reasons. First, most addiction trainees generally have limited opportunities to interact with a high volume of adolescent patients. Simulation scenarios with live actors are ideal for practicing high-stakes human interactions with patients with high acuity or uncommon conditions rarely encountered in clinical practice.[18] Second, simulation with live actors allows trainees to practice nuanced interpersonal skills needed to provide effective counseling and referral to treatment for pediatric patients. Finally, the ability to debrief with expert faculty and reflect on one's performance facilitates learner-driven education and is a key piece of simulations. Our goal was to use dynamic interactions with a live actor to recreate the realistic challenges innate to discussing a sensitive topic with a pediatric patient and to help our trainees increase their confidence and self-efficacy in providing evidence-based treatment options for adolescent OUD.

Methods

Development

We developed a simulation-based educational intervention (Appendix A) for the management of adolescents with OUD and implemented the curriculum with subspecialty trainees in both addiction medicine and addiction psychiatry. We gathered participant demographic information (Appendix B), evaluated the curriculum with pre- and postsimulation confidence surveys (Appendix C), and used a critical actions checklist (Appendix D) as a formative assessment to support faculty during the debrief. This curriculum evaluation was approved as an exempt study by our university's institutional review board.

We created the simulation-based educational intervention in an interdisciplinary collaboration between addiction specialists, pediatricians, and medical simulation experts. Prior to implementing the simulation-based intervention, we pilot tested the simulation with an addiction medicine physician who had recently completed specialty training. The graduate provided feedback, which was incorporated into the final product. Once the curriculum had been developed, we incorporated it into addiction psychiatry and addiction medicine fellows' first 6 months of subspecialty training. All trainees were X/Drug Addiction Treatment Act waivered; however, most participants had limited experience working with adolescents in clinical settings and less in working with adolescent substance use (Table 1). All had been trained in the diagnosis of OUD, treatment options for OUD in adults, and identification of signs and symptoms of opioid withdrawal. To participate in this course, we

Copyright © 2021 Garcia-Vassallo et al. This is an open-access publication distributed under the terms of the Creative Commons Attribution license.

Table 1. Participants' Demographic Information (*N* = 35)

Item	No. (%)
Completed assessments	
Pretest	35 (100)
Posttest	25 (71)
Gender	
Female	14 (40)
Male	21 (60)
Race (select all that apply)	
White	22 (63)
Black	5 (14)
Asian/Pacific Islander	1 (3)
Other	8 (23)
Prefer not to answer	2 (6)
Ethnicity	
Hispanic/Latinx	5 (14)
Total individuals with additional experience in adolescent substance abuse disorder	7 (20)
Elective rotations during residency	5 (14)
Elective rotations during fellowship	2 (6)
As an attending	2 (6)
Elective online training	2 (6)
Elective conferences	4 (11)
Other	1 (3)

recommended that trainees have basic background knowledge on the diagnosis and management of OUD as well as on symptomatology associated with opioid withdrawal. X waiver training was not required to participate.

Overview and Preparation

The simulation experience lasted approximately 60 minutes, with time allotted for trainees to review their learner packet case materials (Appendix E), receive a brief introduction prior to the simulation scenario, undergo the simulation itself, complete a self-reflection, debrief with faculty, and participate in a postsimulation satisfaction survey (Appendix F).

Prior to implementation, we created a video recording of the PowerPoint titled "Management of Adolescents With Opioid Use Disorder" (Appendix G). The video described clinical situations wherein adolescents had the right to confidentiality and offered strategies on how to communicate this information to adolescent patients. In addition, it reviewed withdrawal symptoms associated with OUD and evidence-based medication treatment for OUD in adolescents. (We include the PowerPoint version of the presentation for ease of adaptation to other settings, where a new recording will need to be made.) We sent the 10-minute video to trainees 1 week prior to the simulation for them to review as prework.

Standardized patients (SPs), composed of both professional actors and volunteer students from other departments of the university, received the SP packet (Appendix H), which included the simulation scenario and objectives along with SP background material and an opioid withdrawal symptom cheat sheet, prior to the simulation. The background material and cheat sheet provided the SPs with specific instructions regarding responses to examinees' statements, including scripted responses to potential statements. One of the facilitators met individually with the SP actor prior to the simulation and reviewed the material, schedule, stated objectives, and symptoms of opioid withdrawal. A second meeting was held at the conclusion of the simulations to debrief and elicit feedback from the actor.

Facilitators were trained addiction specialists, and one had additional expertise in working with adolescents. To offer this course elsewhere, we recommend that facilitators have some background knowledge of the management of OUD and be familiar with local state laws regarding adolescents. Included in Appendices G and I is a link to a recent review article on medications for OUD in adolescents (prereading). In addition, some helpful internet links on adolescents' consent and confidentiality can be found at the conclusion of the PowerPoint slides, along with a link for modules on addiction treatment. Finally, the facilitator's debriefing guide (Appendix I) highlights all major learning points and can be used as a guide during the facilitator-learner debrief.

Equipment/Environment

The scenario took place in a simulated patient room resembling an outpatient evaluation room within a medical school–based immersive health care simulation center. The debriefing session was conducted in a separate confidential conference room. During the simulation, the SP used a cellular phone as a prop, and a chair was provided for the medical trainee. A facilitator stood in the control room next door behind a one-way mirror and offered one-way instructions through a handheld transceiver to the SP's earpiece. SPs received a packet (Appendix H) with the learning objectives, simulation scenario, cheat sheet of withdrawal symptoms, and background information prior to the exercise and had the opportunity to discuss the material in detail with facilitators. We trained and coached SPs on how to appear in opioid withdrawal and reviewed prompted responses ("Are you going to tell my Mom?") to elicit trainee learning objectives. The length of time devoted to SPs' training varied based on their prior knowledge and experience regarding the topic and ranged anywhere from 20 to 40 minutes. Upon arrival and prior to the simulation, trainees received the learner packet (Appendix E), which included the simulation-based learning philosophy, case description, learning objectives, patient vital signs, urine toxicology results, Clinical Opiate Withdrawal Scale,

Copyright © 2021 Garcia-Vassallo et al. This is an open-access publication distributed under the terms of the Creative Commons Attribution license.

and self-debrief. The packet was available for trainees to use as a reference throughout the exercise.

Personnel

To successfully use this case scenario, implementers will likely need at least one trained facilitator and an SP. In our situation, we had three trained facilitators in order to create efficiency and facilitate parallel, concurrent sessions for multiple learners. One facilitator oriented the trainees upon arrival and provided an exit interview. The other two facilitators were paired one to one with a trainee for the simulation/debrief, alternating their use of the simulation and debrief room. Learners participated in the simulation individually and were debriefed by the facilitators one-on-one. An SP who appeared young enough to resemble a teenager portrayed the adolescent patient for all the simulations within each given cohort. In addition, we had a simulation technician who helped set up equipment. Depending on a facility's available resources, the technician role could be filled by the facilitator.

Implementation

We invited addiction psychiatry and addiction medicine fellows to participate in the educational activity. Fellows were expected to attend the simulation and debrief as part of their addiction training curriculum; however, surveys were optional, and fellows could opt out of survey/data collection. One week prior to the simulation, trainees completed an electronic survey to measure their confidence in learning topics. Subsequently, learners participated in the presimulation educational didactic, which included (1) viewing a 10-minute PowerPoint video addressing learning objectives and (2) completing a baseline survey describing previous relevant clinical experience and basic demographic information. Trainees received a scheduled time and directions to the simulation center via email. On the day of the simulation program, a facilitator greeted trainees with the learner packet and answered initial questions before escorting them individually to the entrance of the simulated outpatient office. Once inside, the trainee interacted with the SP actor in a 20-minute simulated case encounter featuring a treatment-seeking adolescent with OUD in early signs of opioid withdrawal. Participants assessed the signs and symptoms of opioid withdrawal and explained treatment options and confidentiality to the adolescent. A facilitator stood in the control room next door behind a one-way mirror, offered one-way instructions through a handheld transceiver to the SP's earpiece, and completed the critical actions checklist (Appendix D). After the simulation, participants were given some time to reflect on the experience and complete the self-debrief sheet included in their learner packet, followed by an in-person debriefing session with their facilitator. Facilitators utilized the participant's self-debrief sheet, critical action checklist results, and debrief guide to facilitate the discussion, review performance, and exchange impressions. During the debrief, trainees identified three learning goals and wrote these down in their packets. Trainees completed a satisfaction survey at the end of the activity and a posttraining survey to measure retained confidence 1 month following the exercise. Facilitators also met with the SP at the end of the exercise to elicit feedback and debrief on their experience.

Debriefing

At the conclusion of the 20-minute simulation, learners were given some time to reflect and complete a self-reflection sheet, which served as a tool to initiate the process of reflective learning. The facilitator-learner debriefing session merged various educational strategies into a blended debriefing framework to maximize learner engagement.[19] We followed the overall structure of the PEARLS (Promoting Excellence and Reflective Learning in Simulation) framework to develop our debriefing.[19] First, the facilitator reviewed the learners' self-debrief and evoked participants' emotional reactions, revealing key issues important to the learners. This allowed a common agenda to develop. Participants then summarized the case, with the goal of highlighting their understanding of the learning objectives or lack thereof. Next, learner frames were evoked with the use of the advocacy/inquiry technique[20] to probe for particular learner actions or challenges that facilitators had noted as critical learning points during the simulation. The facilitator then guided participants through critical reflection and delivered key learning points through focused teaching with the use of the debrief guide. Directions for future learning were developed in collaborative dialogue between learner and facilitator, and three learning goals were identified. In addition to the debrief guide, facilitators utilized a recent review on medication management for adolescents with OUD in preparation for the debrief (prereading document, link in Appendices G and I).[21]

Assessment

To measure the effectiveness of the simulation in achieving the stated educational objectives, learners completed a confidence survey 1 week prior to and again 1 month following the training to quantify changes in confidence. We modified previously developed confidence scales measuring these constructs in smoking and lipid intervention trials into a seven-item questionnaire to measure changes in confidence for specific

Copyright © 2021 Garcia-Vassallo et al. This is an open-access publication distributed under the terms of the Creative Commons Attribution license.

tasks.[22,23] Participants completed a self-reflection in which they summarized details of what had happened during the simulation and identified future learning goals. We assessed participant satisfaction with a three-item survey aimed at capturing participant level of comfort, perceived effectiveness, and probability to recommend the exercise (Appendix F). We used a critical actions checklist (Appendix D) for formative assessment and support for the debrief. Our critical actions checklist included major learning points derived from our objectives.

Results

Between 2016 and 2019, a total of 35 fellows (21 male, 14 female) participated in the educational activity. Seven of the participants reported having received some training on adolescents with substance use disorders prior to the exercise. Prior reported training consisted of elective clinical rotations, elective online courses, and attendance at conferences, among others (Table 1). Of the participants, 100% (35) completed the confidence presurvey and 71% (25) the postsurvey. Fellows filled out a six-item questionnaire, rating each item on a 10-point Likert scale, to self-assess their level of confidence completing each of six designated tasks during the simulation (Table 2). Paired *t*-test results of pre-/postsession assessments of confidence resulted in an overall increase of confidence scores for all tasks, with "the ability to describe available treatment options in the state of Connecticut for adolescents with OUD" demonstrating the biggest change in confidence. Of the 26 participants who completed confidential satisfaction surveys, 88% (23) said they felt comfortable during the training, 96% (25) said they found the exercise effective, and 100% (26) said they would recommend the training to others.

At the conclusion of the simulation exercise, trainees completed a self-debrief that asked two questions: How did you feel about the case? Can you summarize what happened during the case? All participants were able to summarize the case scenario and the three main learning objectives. Recurrent themes describing feelings about the case included the following (the number of learners who described the theme is given in parentheses at the end of each item):

- Allows for deliberate practice: "The simulation was a great way to practice communicating with an adolescent patient" (14).
- Anxiety: "I felt anxious because: lack of experience with adolescents" (four), "lack of medical knowledge" (five), "being watched/evaluated" (two), "didn't know what to expect" (one).
- Communicating with adolescents: "Talking to an adolescent is challenging" (six).
- Realistic: "The case felt real and was reasonable" (six).

Finally, after completing the debrief with a facilitator, learners identified future learning goals. Recurrent learning goals that emerged included the following (the number of learners who described the learning goal is given in parentheses at the end of each item):

- Continuing to practice communicating medical knowledge and establishing rapport with adolescents (15).
- Identifying treatment options for adolescents in the community I practice/will practice (11).
- Reading further on medications and behavioral interventions for adolescents with OUD (11).
- Researching federal guidelines and policies surrounding methadone treatment for adolescents (eight).
- Reviewing statutes of confidentiality in different states/countries (seven).
- Engaging family in treatment for adolescents with OUD (five).

Table 2. Participants' Confidence in Working With Adolescents With OUD (*N* = 35)

Item[a]	Pretest Confidence: *M* (*SD*)	Posttest Confidence: *M* (*SD*)	Change in Confidence: *M* (95% CI)	*P*[b]
Establish rapport with the adolescent.	6.8 (1.5)	8.2 (1.1)	1.4 (0.7-2.0)	<.001
Explain the limits of confidentiality.	6.2 (1.5)	8.1 (1.7)	1.9 (1.1-2.7)	<.001
Identify opioid withdrawal symptoms using the Clinical Opioid Withdrawal Scale.	7.9 (1.8)	8.7 (1.1)	0.8 (0.1-1.4)	.025
Rate the severity of opioid withdrawal as mild, moderate, severe.	7.5 (1.6)	8.5 (1.2)	1.0 (0.4-1.7)	.003
Describe at least three available treatment options in the state of Connecticut for adolescents with OUD.	4.8 (2.7)	7.8 (2.1)	3.0 (2.0-4.0)	<.001
Determine whether it is appropriate to inform the parent/guardian of substance use behaviors.	5.7 (1.8)	7.8 (1.6)	2.1 (1.1-3.1)	<.001

Abbreviation: OUD, opioid use disorder.
[a]Confidence rated on a 10-point Likert scale (1 = *Cannot Do at All,* 10 = *Highly Certain Can Do*).
[b]Significant at $p < .05$.

Copyright © 2021 Garcia-Vassallo et al. This is an open-access publication distributed under the terms of the Creative Commons Attribution license.

Discussion

This step-by-step description of our activity demonstrates how to implement a simulation-based educational intervention on management of adolescents with OUD. At the time of writing, no peer-reviewed simulation-based curricula addressing the management of adolescents with OUD, a debilitating chronic condition that often begins during this vulnerable developmental stage in which concerns about privacy influence health care decisions and ability to openly communication with health care professionals,[7] were known to have been published. The lack of opportunities during training to manage adolescents with OUD make our simulation-based curriculum ideal for this rare but critical health scenario. Our curriculum provides learners with an opportunity to demonstrate understanding of confidentiality laws as they pertain to substance use care in adolescents, assess for the presence of opioid withdrawal, and explain age-appropriate medication treatment options to adolescents with severe OUD.

The exercise for subspecialty trainees was feasible, was well received, and increased confidence in all areas as evidenced by an increase in average mean scores in all domains. Most participants felt comfortable throughout, found the exercise effective, and stated that they would recommend the curriculum to others. Although some participants reported experiencing some form of anxiety (e.g., anticipatory or performance related), they also felt the simulation was a great tool to practice engaging with, developing rapport with, and communicating medical knowledge about OUD to an adolescent. Learning goals were identified by all participants, demonstrating the exercise's ability to inspire continued learning and inquiry.

Although our training was conducted in an academic institution with a simulation center, it may be used by training programs without a simulation center, as there is no specific equipment required during the session. The use of a one-way mirror and handheld transceiver (walkie-talkie) is helpful but not necessary for successful execution. Developing rapport with an adolescent and effectively communicating medical knowledge and recommendations often require practice. The simulated case should be portrayed by a young adult to keep the experience as realistic as possible and mimic the discomfort that naturally occurs when counseling an adolescent. Training surrounding OUD and opioid withdrawal symptom manifestation may be necessary for young actors serving as SPs. In addition, postsimulation debriefs are encouraged to answer questions or respond to feelings from the SP that may arise during the interventions. For example, one of our actors shared a story of a friend with opioid addiction and the feelings of empathy for her friend that emerged during the exercise.

Even though this case was developed for addiction psychiatry and addiction medicine subspecialty trainees, it could potentially be adapted for application in other specialties, including general psychiatry, child and adolescent psychiatry, pediatrics, emergency medicine, and obstetrics and gynecology. The training could also be used with other professions, such as nursing, physician assistants, and clinical psychology. Tailoring the curriculum for nonaddiction trainees may require additional prework, such as material that recognizes addiction as a chronic medical condition with available lifesaving medications, as well as guidelines on how to diagnose OUD. The information provided to participants in the PowerPoint should be tailored to reflect state-specific confidentiality laws. Information about state-specific confidentiality laws can be found online through state-specific websites or at the Center for Adolescent Health & the Law.[24]

Limitations of our study include a small sample size of trainees at one training site. However, with most addiction medicine and addiction psychiatry fellowship programs graduating only one to two fellows per year, our sample size is comparatively large. Some participants did not complete the 1-month follow-up or completed it after the deadline. The evaluation did not allow us to determine whether the increase in confidence would translate into improved clinical competence or patient outcomes in real-world clinical settings. In addition, we were unable to discern what led to an increase in confidence scores: the prework, the simulation, or the combination of the two. Future studies should examine these results further by comparing confidence scores of individuals who complete the prework and individuals who do not. We were fortunate to have a pediatrician and psychiatrist, both with addiction expertise, evaluate and debrief participants. The expertise of the faculty may not be generalizable to other settings. However, we believe that nonaddiction experts could implement the curriculum by reviewing local state laws on confidentiality along with other material included in our curriculum (see prereading document[21] links in Appendices G and I). In addition, it would be useful to evaluate this curriculum in a setting outside of a simulation center or via a synchronous online learning platform to assess ease of implementation and generalizability. Lastly, we recognize our exercise does not focus on incorporating family into the management of adolescents with OUD. This topic deserves further discussion in additional educational modules.

Copyright © 2021 Garcia-Vassallo et al. This is an open-access publication distributed under the terms of the Creative Commons Attribution license.

We continue to use this educational activity with incoming addiction medicine and addiction psychiatry fellows at our institution. In response to some of the fellows' identified learning goals, we have begun offering additional resources and learning material at the conclusion of the exercise, such as a detailed manual of local community resources. Future directions may include implementing the curriculum virtually and with other subspecialties, specialties, and health care professionals as well as expanding to national forums. In summary, we have found that a simulation-based educational intervention is an effective teaching method for the management of adolescents with OUD and that this curriculum can help advance the state of opioid education among health care professional trainees.

Appendices

A. OUD Simulation Case.docx
B. Demographic Information Survey.docx
C. Confidence Survey.docx
D. Critical Actions Checklist.docx
E. Learner Packet.docx
F. Learner Satisfaction Survey.docx
G. Management of Adolescents With OUD.ppt
H. Standardized Patient Packet.docx
I. Debriefing Guide.docx

All appendices are peer reviewed as integral parts of the Original Publication.

Gabriela Garcia-Vassallo, MD: Assistant Professor, Department of Psychiatry, Yale School of Medicine; ORCID: https://orcid.org/0000-0002-1095-7493

Ellen Lockard Edens, MD, MPE: Associate Professor and Associate Fellowship Director for Addiction Psychiatry, Department of Psychiatry, Yale School of Medicine

Brady Heward, MD: Assistant Professor, Department of Psychiatry, Robert Larner, M.D., College of Medicine at the University of Vermont; Clinical Instructor, Department of Psychiatry, Yale School of Medicine

Marc A. Auerbach, MD, MSci: Associate Professor, Departments of Emergency Medicine and Pediatrics, Yale School of Medicine

Ambrose H. Wong, MD, MSEd: Assistant Professor, Department of Emergency Medicine, and Associate Fellowship Director, Medical Simulation, Yale School of Medicine

Deepa Camenga, MD, MHS: Associate Professor, Departments of Emergency Medicine and Pediatrics, Yale School of Medicine

Acknowledgments
We would like to thank Curtis W. Bone, MD, and Caroline Falker, MD, for their contributions to this project; Joy Grabow and all the staff at the Yale Center for Medical Simulation for their support; and the Addiction Psychiatry and Addiction Medicine Fellowship classes of 2016-2020 for their participation and feedback.

Disclosures
None to report.

Funding/Support
None to report.

Ethical Approval
The Yale University Institutional Review Board approved this study.

References

1. Gaither JR, Shabanova V, Leventhal JM. US national trends in pediatric deaths from prescription and illicit opioids, 1999-2016. *JAMA Netw Open*. 2018;1(8):e186558. https://doi.org/10.1001/jamanetworkopen.2018.6558
2. Gaither JR, Leventhal JM, Ryan SA, Camenga DR. National trends in hospitalizations for opioid poisonings among children and adolescents, 1997 to 2012. *JAMA Pediatr*. 2016;170(12): 1195-1201. https://doi.org/10.1001/jamapediatrics.2016.2154
3. Hadland SE. How clinicians caring for youth can address the opioid-related overdose crisis. *J Adolesc Health*. 2019;65(2): 177-180. https://doi.org/10.1016/j.jadohealth.2019.05.008
4. Hadland SE, Bagley SM, Rodean J, et al. Receipt of timely addiction treatment and association of early medication treatment with retention in care among youths with opioid use disorder. *JAMA Pediatr*. 2018;172(11):1029-1037. https://doi.org/10.1001/jamapediatrics.2018.2143
5. Madras BK, Ahmad J, Wen J, Sharfstein J; Prevention, Treatment, and Recovery Working Group of the Action Collaborative on Countering the U.S. Opioid Epidemic. Improving access to evidence-based medical treatment for opioid use disorder: strategies to address key barriers within the treatment system. *National Academy of Medicine*. 2020. https://doi.org/10.31478/202004b
6. Andraka-Christou B, Capone MJ. A qualitative study comparing physician-reported barriers to treating addiction using buprenorphine and extended-release naltrexone in U.S. office-based practices. *Int J Drug Policy*. 2018;54:9-17. https://doi.org/10.1016/j.drugpo.2017.11.021
7. Ford CA, Bearman PS, Moody J. Foregone health care among adolescents. *JAMA*. 1999;282(23):2227-2234. https://doi.org/10.1001/jama.282.23.2227
8. Talib HJ, Silver EJ, Alderman EM. Challenges to adolescent confidentiality in a children's hospital. *Hosp Pediatr*. 2016;6(8):490-495. https://doi.org/10.1542/hpeds.2016-0011

Copyright © 2021 Garcia-Vassallo et al. This is an open-access publication distributed under the terms of the Creative Commons Attribution license.

9. Tesema L, Marshall J, Hathaway R, et al. Training in office-based opioid treatment with buprenorphine in US residency programs: a national survey of residency program directors. *Subst Abus*. 2018;39(4):434-440. https://doi.org/10.1080/08897077.2018.1449047
10. Vettese TE, Thati N, Roxas R. Effective chronic pain management and responsible opioid prescribing: aligning a resident workshop to a protocol for improved outcomes. *MedEdPORTAL*. 2018; 14:10756. https://doi.org/10.15766/mep_2374-8265.10756
11. Aultman W, Fett J, Lauster C, Muench S, Halalau A. Urine drug test interpretation: an educational program's impact on resident knowledge and comfort level. *MedEdPORTAL*. 2018;14:10684. https://doi.org/10.15766/mep_2374-8265.10684
12. Jawa R, Luu T, Bachman M, Demers L. Rapid naloxone administration workshop for health care providers at an academic medical center. *MedEdPORTAL*. 2020;16:10892. https://doi.org/10.15766/mep_2374-8265.10892
13. Derefinko KJ, Brown R, Danzo A, et al. Addiction medicine training fellowships in North America: a recent assessment of progress and needs. *J Addict Med*. 2020;14(4):e103-e109. https://doi.org/10.1097/ADM.0000000000000595
14. Bowyer MW, Hanson JL, Pimentel EA, et al. Teaching breaking bad news using mixed reality simulation. *J Surg Res*. 2010;159(1):462-467. https://doi.org/10.1016/j.jss.2009.04.032
15. Sukalich S, Elliott JO, Ruffner G. Teaching medical error disclosure to residents using patient-centered simulation training. *Acad Med*. 2014;89(1):136-143. https://doi.org/10.1097/ACM.0000000000000046
16. Barsuk JH, Cohen ER, Williams MV, et al. Simulation-based mastery learning for thoracentesis skills improves patient outcomes: a randomized trial. *Acad Med*. 2018;93(5):729-735. https://doi.org/10.1097/ACM.0000000000001965
17. Ahya SN, Barsuk JH, Cohen ER, Tuazon J, McGaghie WC, Wayne DB. Clinical performance and skill retention after simulation-based education for nephrology fellows. *Semin Dial*. 2012;25(4): 470-473. https://doi.org/10.1111/j.1525-139X.2011.01018.x
18. Lewis KL, Bohnert CA, Gammon WL, et al. The Association of Standardized Patient Educators (ASPE) Standards of Best Practice (SOBP). *Adv Simul (Lond)*. 2017;2:10. https://doi.org/10.1186/s41077-017-0043-4
19. Eppich W, Cheng A. Promoting Excellence and Reflective Learning in Simulation (PEARLS): development and rationale for a blended approach to health care simulation debriefing. *Simul Healthc*. 2015;10(2):106-115. https://doi.org/10.1097/SIH.0000000000000072
20. Rudolph JW, Simon R, Dufresne RL, Raemer DB. There's no such thing as "nonjudgmental" debriefing: a theory and method for debriefing with good judgment. *Simul Healthc*. 2006;1(1):49-55. https://doi.org/10.1097/01266021-200600110-00006
21. Camenga DR, Colon-Rivera HA, Muvvala SB. Medications for maintenance treatment of opioid use disorder in adolescents: a narrative review and assessment of clinical benefits and potential risks. *J Stud Alcohol Drugs*. 2019;80(4):393-402. https://doi.org/10.15288/jsad.2019.80.393
22. Ockene JK, Quirk ME, Goldberg RJ, et al. A residents' training program for the development of smoking intervention skills. *Arch Intern Med*. 1988;148(5):1039-1045. https://doi.org/10.1001/archinte.1988.00380050045007
23. Ockene JK, Ockene IS, Quirk ME, et al. Physician training for patient-centered nutrition counseling in a lipid intervention trial. *Prev Med*. 1995;24(6):563-570. https://doi.org/10.1006/pmed.1995.1090
24. Consent & confidentiality protection. Center for Adolescent Health & the Law. Accessed March 3, 2021. https://www.cahl.org/publications/consent-confidentiality-protection/

Received: July 27, 2020
Accepted: February 22, 2021
Published: April 20, 2021

Copyright © 2021 Garcia-Vassallo et al. This is an open-access publication distributed under the terms of the Creative Commons Attribution license.
